# A Bacterial Kind of Aging

**DOI:** 10.1371/journal.pgen.0030224

**Published:** 2007-12-14

**Authors:** Thomas Nyström

**Affiliations:** Baylor College of Medicine, United States of America

Bacteria are sometimes honored with a few lines in books and reviews on aging as an example of organisms that do not age. This is because binary fission of bacteria has been assumed to proceed with a nonconservative dispersion of both undamaged and damaged constituents, such that there are no adult forms of bacterial cells and the bacterial population is not age structured. However, some authors have expressed different views; for example, Partridge and Barton [1] consider asymmetry in simple unicellular systems and how this might develop into aging, and Tom Kirkwood [2] argues, on theoretical grounds, that damage segregation could be selected for in simple unicellular systems dividing by binary fission, and that sibling-specific deterioration may confer a selective advantage. Indeed, recent reports lend experimental support to this notion and point to mandatory aging also being a part of the life history of bacteria.

These reports include the demonstration that cells of an Escherichia coli population exhibit markedly different loads of damaged proteins [3]. This damage heterogeneity does not follow a simple normal distribution but rather indicates that the population consists of two discrete populations with respect to damage; a damage-enriched and a low-damage population [3]. Moreover, the low-damage cells remain reproductively competent, whereas damage-enriched cells become genetically dead (non-culturable) [3]. In addition, and most importantly, bacterial cells have been shown to exhibit signs of replicative aging, or loss of fitness, in a sibling-specific manner during exponential growth; i.e., a cumulative loss of fitness in one sibling lineage that could be argued to serve as a “mother-type” lineage, similar to that of the budding yeast Saccharomyces cerevisiae [4,5].

## Evidence of Mandatory Bacterial Aging

Caulobacter crescentus, in which cytokinesis is intrinsically asymmetrical, was the first bacterium reported to exhibit replicative aging [4]. In this bacterium, a stalked cell generates a motile swarmer cell, which, after differentiation into a stalked cell, can itself give rise to progeny. However, with each division, the stalked cell requires progressively longer times to produce a swarmer cell, a manifestation of replicative aging [4]. The second bacterium reported to show signs of replicative aging was E. coli, an organism that divides by binary fission, and, as far as we know, lacks a sibling-specific differentiation. By tracking the poles of E. coli cells and measuring the cells' increases in length during growth, it was possible to calculate the generation time of individual cells [5]. By doing so, the authors found that the growth rate decreases in cells inheriting old poles, suggesting that E. coli cells, like C. crescentus and S. cerevisae, are subjected to lineage-specific replicative aging [5]. Prior to the study on E. coli, Barker and Walmsley [6] demonstrated that a eukaryotic organism, Schizosaccharomyces pombe, dividing by symmetrical binary fission, also shows signs of replicative aging.

Thus, the accumulated data from different unicellular models suggest that a sibling-specific reduction in fitness (growth rate) may be more common than previously anticipated and that cytokinesis during binary fission is inherently asymmetrical. But can we extrapolate the data and assume that the reduction in sibling-specific growth rate will eventually cause the cell to die? Woldridge [7] argued against such a conclusion, because taking the variabilities of E. coli cell length and age at division into account, the sibling-specific decreases in growth rate fall within the expected variation, and are sufficiently different from the catastrophe-like cell death arrived at through replicative aging. However, the growth rate of old-pole E. coli cells becomes successively slower during the divisions studied [5,8], and it would be almost impossible to carry the experiment out long enough to get statistically significant data on sibling-specific cell death in the system employed. Regardless of whether the system eventually reaches a catastrophe or a steady state, the progressive reduction in sibling-specific growth rate is highly intriguing because it raises questions regarding the ultimate and proximate causation of fitness asymmetry in a unicellular system.

## Ultimate Causation for Asymmetry

Is there an advantage to producing daughter cells of unequal reproductive potential or is asymmetry caused by accidental, physical, or metabolic constraints that have no obvious bearing on fitness? In an attempt to elucidate the pros and cons of symmetrical and asymmetrical bacterial division, Watve et al. [9] modeled growth and the propagation of growth-limiting components of a unicellular system using a modified Leslie matrix framework. As developed, the model points to asymmetrical division favoring rapid growth, whereas symmetry results in slow growth but higher efficiency; i.e., a higher growth yield [9]. Similarly, using an individual-based simulation approach, Ackermann et al. [10] found that a differentiation between an aging parental cell and a rejuvenated progeny readily evolves to cope with self-inflicted damage. Johnson and Mangel [11], using the Euler-Lotkas equation, came to a similar conclusion. In addition, asymmetrical segregation of damage that cannot be repaired may be beneficial at high cell densities and slow rates of replication [12]. Also, upon transient external stresses reaching lethal levels, an asymmetrical segregation of irreparable damage may permit survival of the clone at the expense of the “mother-type” cells, in which the damage is retained [13].

Thus, very different types of models and simulations suggest that sibling-specific asymmetry may provide the system with a fitness advantage and that replicative aging evolved early in the history of life [10]. However, at present, the models and simulations are hampered by the fact that we know very little about the nature of the critical components (aging factors) reducing cellular fitness and the mechanisms establishing their asymmetrical distribution. Elucidating these features will be critical in estimating the energetic costs for damage segregation versus damage removal (assuming that damage is, at least partly, responsible for bacterial aging) and why segregation might, in some cases, be selected over damage repair/removal.

## Proximate Causation for Asymmetry

One common assumption in the reports modeling potential benefits of asymmetry is that the establishment of age asymmetry is linked to damage segregation [9–13]. The question that arises is, what kind of damaged, or toxic, molecules are critical in affecting sibling-specific fitness? In E. coli, is it the old pole itself, the parental DNA strand segregating to the old pole [14], damaged and cytotoxic DNA molecules predominantly inherited by the old pole cell [15], or some deteriorated and potentially cytotoxic molecules, such as protein aggregates, in the cytoplasm (Figure 1)?

**Figure 1:**
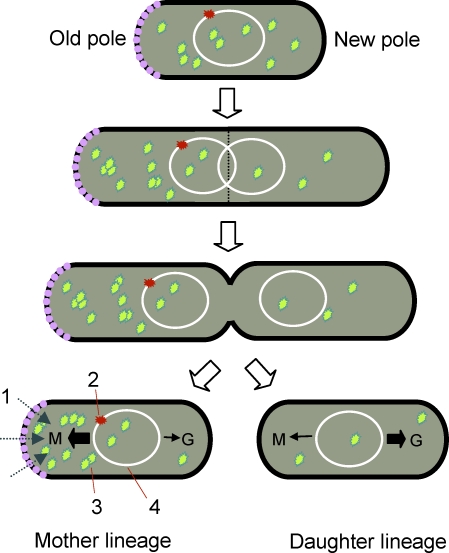
Schematic Representation of Possible Aging Factors in a System Dividing by Binary Fission During cytokinesis, one daughter cell will inherit a pole that is older than the one inherited by its sibling. Intriguingly, the “old pole” cells of E. coli display a progressive increase in their generation time [5]. There are several potential reasons for this decline in physiological fitness: (1) Inheritance of older cell-surface material may reduce the ability of the cell to insulate itself against the environment. (2) Segregation of differently damaged, and potentially cytotoxic, DNA strands [15] could provide one daughter with a noncorrupt message akin to the “immortal DNA strand” cosegregation mechanism originally proposed by Cairns for preserving the integrity of stem cell genomes [27]. (3) Segregation of cytotoxic molecules, such as extragenomic episomes or oxidatively damaged and aggregated proteins, may result in sibling-specific deterioration. (4) Segregation of damage could cause a reduction in fitness even in the absence of cytotoxicity, since the sibling inheriting more damage may, as a consequence, upregulate maintenance (M) (damage defense) systems. In view of the fact that the transcriptional power of cells like E. coli is limiting, such an elevation of maintenance activities could be traded for a reduction of growth-related activities (G) [28,29].

In budding yeast, cytotoxic extrachromosomal rDNA circles and oxidatively damaged proteins are segregated such that the mother cell retains most of these molecules during cytokinesis [16,17]. The yeast anti-aging protein Sir2p governs the management of both extrachromosomal rDNA circles and oxidatively damaged proteins [16,17], and a model for the Sir2p-dependent retention of oxidatively damaged proteins was recently presented, involving the aggregation-remodeling factor Hsp104p in concert with the actin cytoskeleton [18]. Interestingly, damage segregation in budding yeast becomes more pronounced following increased oxidative stress [17], suggesting that the efficiency of damage segregation is not fixed in this species but can be adjusted with changing environmental demands. This raises the question of whether replicative aging in the bacterial systems studied becomes more or less pronounced depending on growth conditions; for example, during growth at different oxygen tensions or on plates containing antioxidants.

Stationary-phase die-off of S. cerevisiae cells (sometimes referred to as chronological aging) has been firmly linked to oxidative damage and genetic alterations affecting reactive oxygen species production and scavenging are effective in retarding stationary phase death in this model system [19–22]. Likewise, self-inflicted oxidative damage has been implicated in cellular degeneration of stationary-phase bacteria [23–25], and a recent report showed that three different classes of bactericidal antibiotics, regardless of their drug–target interactions, cause bacterial cell death by stimulating the production of highly deleterious reactive oxygen species [26]. Thus, it would be of great interest to learn whether oxidatively damaged (aggregated) molecules are segregated during bacterial cytokinesis, and if they, indeed, act as bona fide aging factors.

However, one should not put all of one's eggs in the same basket; indeed, one of the most exciting features of the discovery of a mandatory aging phenomenon in bacteria and eukaryotes dividing by binary fission is that, by virtue of being exquisitely tractable systems for genetic and biochemical analysis, there is a good chance of identifying the true aging agents in these systems. Such knowledge may have an enormous impact on the aging field as a whole.
